# Precarious employment and its relation to mental well-being in the gig economy: comparing main and supplementary workers

**DOI:** 10.5271/sjweh.4278

**Published:** 2026-05-01

**Authors:** Elief Vandevenne, Christophe Vanroelen, Lara Stas, Jessie Gevaert

**Affiliations:** 1 Brussels Institute for Social and Population Studies (BRISPO), Vrije Universiteit Brussel, Brussels, Belgium.; 2 Fonds Wetenschappelijk Onderzoek, Brussels, Belgium.; 3 Core Facility of Statistics, Vrije Universiteit Brussel, Brussels, Belgium.

**Keywords:** employment quality, gig work, job insecurity, occupational health, precarious work, precarity, psychosocial risk factors

## Abstract

**Objectives:**

This study investigates the association between precarious employment (PE) and mental well-being and examines the mediating role of five intrinsic quality of work (IQW) dimensions: autonomy, physical demands, work intensity, skill discretion, and social support. We distinguish between main (>24 hours/week) and supplementary (≤24 hours/week) gig workers.

**Methods:**

Survey data from 397 Belgian gig workers were used to construct a multidimensional, gig-specific measure of PE (the Employment Precariousness Scale for gig work: ‘EPRES-gw’). Structural equation modeling was applied to test mediation by IQW dimensions and examine differences between main and supplementary gig workers.

**Results:**

PE was positively associated with poor mental well-being among main but not supplementary workers. Among main gig workers, the relationship was mediated by high work intensity, physical demands, and low social support. For supplementary workers, low autonomy mediated the association.

**Conclusion:**

PE is particularly relevant for the mental well-being of main gig workers. The role of IQW is important, though the key dimensions differ by time spent in in gig work. These findings highlight the need for policy interventions and research addressing both employment rights and IQW-related psychosocial risks, with approaches tailored to the distinct challenges of main versus supplementary gig workers.

Digital labor platforms mediate transactions between workers and customers, facilitating both online gig work and local services (1), typically without the social rights and protections associated with standard employment relationships (2). Some scholars highlight the potential benefits of gig work, such as flexibility, autonomy, and entrepreneurial opportunities (3), which may support mental well-being via better work–life balance (4, 5). But evidence increasingly points to job insecurity, material deprivation, and limited career prospects among gig workers (5–7), all linked to poorer mental health outcomes (eg 8, 9,).

These dynamics underscore the relevance of precarious employment (PE), a multidimensional construct that captures the accumulation of adverse employment quality characteristics (10, 11). While gig work has been widely studied, quantitative evidence linking PE to gig workers’ mental well-being remains limited (12). Among salaried workers, PE is negatively associated with health and well-being (10, 13), although partly mediated by intrinsic quality of work (IQW) characteristics such as work intensity, physical demands and autonomy (9). Given the task heterogeneity of gig work, similar mediation mechanisms are plausible (14).

Gig work also challenges conventional assumptions of full-time employment. While some engage in gig work as a side hustle (15), for others it entails substantial time engagement (16). The relationship between PE and mental well-being may therefore differ depending on whether gig work is a primary or supplementary activity, as variations in time engagement can influence exposure to PE (12, 17). Analyses of gig workers’ well-being should therefore distinguish between main and supplementary workers.

This study focuses on Belgian gig workers, defined as individuals performing online and/or offline tasks via digital platforms (1). In 2022, about 84 000 people aged 15–64 engaged in ≥1 hour of gig work in Belgium, amounting to roughly 1% of the workforce (18). Gig work is concentrated in transport, household, and professional services, primarily in urban areas (19). Gig workers tend to be highly educated, slightly younger than the general population, and predominantly self-employed (19). Despite its modest scale, gig work increasingly shapes employment relations beyond platforms through performance-based ratings, on-demand scheduling, and digital monitoring in conventional sectors (20, 21). Examining PE in gig work therefore provides broader insights into labor market de-standardization and its implications for worker health. Against this backdrop, this study investigates how PE relates to gig workers’ mental well-being.

## Precarious employment and mental well-being in the gig economy

Since the 1970s, Western labor markets have shifted toward de-standardization, with temporary, interim, and part-time jobs eroding the Fordist model that once provided stability, social protection, and collective bargaining rights (10, 13). Gig work represents an advanced form of this trend and has prompted growing attention to PE as a social determinant of health (13, 22).

Operating within existing legal frameworks (5), gig work amplifies adverse health-related features of PE documented among salaried workers (13, 23). Its expansion after the 2008 financial crisis was fueled by austerity measures and labor market reforms and is characterized by short-term employment, automated task allocation and monitoring, and the absence of a traditional employment relationship (2, 6, 7).

To study PE systematically, researchers have developed multidimensional measures. The Employment Precariousness Scale (EPRES), originally designed for salaried workers in Spain (24), consistently links PE to poor mental health (eg 8, 22,). Few studies have applied it to gig workers with the exception of Vandevenne & Vanroelen (25), who piloted the EPRES-gw with Belgian food couriers and later extended it to gig workers more broadly (26). The EPRES-gw adapts six dimensions for gig workers: (i) temporariness, (ii) disempowerment, (iii) lack of workplace rights, (iv) low wages, (v) lack of fringe benefits, and (vi) lack of training. This preserves consistency with prior EPRES studies (24), while accounting for the specific features of gig work (26).

## The mediating role of intrinsic quality of work

Beyond employment conditions, intrinsic task-related characteristics may represent an additional pathway linking PE to mental well-being. Gig work encompasses a wide range of activities, resulting in substantial variation in working conditions and skill use (1). IQW characteristics such as autonomy, skill discretion, work intensity, physical demands and social support are well-established determinants of mental well-being and have been shown to covary with PE among salaried workers (14, 22).

In gig work, physical demands vary by task type (27). Many platform-mediated jobs operate in weakly regulated health and safety contexts (6), exposing workers to physical strain that may be exacerbated by piece-rate pay systems (28), which can negatively impact well-being (29). By contrast, online gig work is typically less physically demanding, and work intensity tends to be lower among those who engage only occasionally (30). Autonomy and skill discretion also differ widely: algorithmically managed, short-cycle tasks may constrain skill use and undermine well-being (2, 6, 31), whereas more complex and autonomous tasks can support skill development and mental health (32). Finally, social support is often constrained by digital intermediation, reducing contact with co-workers, fostering isolation (28, 29) or competition (33), although in some sectors – such as food delivery or ride-hailing – regular encounters among workers are more common (34). Extended working hours may further intensify these effects (6).

In sum, IQW dimensions likely mediate the association between PE and mental well-being (8, 9, 22).

## Explaining variations in mental well-being between main and supplementary gig workers

A defining feature of the gig economy is the shift from job- to task-based work. A 2022 European Trade Union Institute study estimated that around three-quarters of gig workers participate only occasionally, while approximately one-quarter rely on gig work as their main activity (16). Distinguishing between these groups is essential when assessing the mental well-being implications of PE (12, 17).

Evidence indicates that greater time spent in gig work is associated with increased exposure to precarious conditions and poorer mental well-being (12, 16, 35). Main gig workers report lower flexibility, greater difficulty securing sufficient income, and fewer opportunities to avoid undesirable tasks (17). Their higher time investment likely amplifies the adverse effects of PE, including heightened vulnerability to income and contractual insecurity (17). By contrast, supplementary gig workers often retain economic and social security through other roles – such as wage employment or education – and may perceive gig-related risks as less consequential (12, 17). This relative security also facilitates disengagement and reduces incentives for collective action (34).

Accordingly, this study examines the relationship between PE and mental well-being among gig workers, accounting for the mediating role of IQW and differences between main and supplementary gig workers. Using the adapted EPRES-gw and survey data from 397 Belgian gig workers, we employed structural equation modeling (SEM) to assess: (i) the direct association between PE and mental well-being, hypothesizing that higher PE is linked to poorer mental well-being, with stronger effects among main gig workers, and (ii) whether IQW dimensions—autonomy, work intensity, physical demands, skill discretion, and social support—partially mediate the relationship between PE and mental well-being, and whether these mediation effects are more pronounced for main gig workers.

## Methods

### Data

We used cross-sectional survey data collected in 2022–2023 (26) among Belgian gig workers, covering working conditions, health, mental well-being, employment status, and socio-demographics. All gig workers who had engaged in gig work at least once in the past six months were eligible, regardless of the type of work. In the final sample, the types of gig work that was represented included food delivery and transport, other on-location tasks (eg, tutoring, childcare, dog walking, handymen chores), micro-tasks, and online professional/ administrative tasks. Respondents were recruited via multiple non-probability sampling strategies to reach maximum variation in types of work, including referral through representatives of trade unions and platform companies, social media advertisements, web panels, direct messaging through the platforms, and in-person recruitment of food couriers and taxi drivers [see (36) for details]. The combined sample included 528 participants (397 self-recruited, 131 from an online panel). After excluding cases with less than two-thirds of scale responses completed, the final sample comprised 397 respondents. A structural comparison between the self-recruited and panel respondents (36) indicated that both could be combined, with only minor differences: panel respondents were slightly more likely to be female, older, highly educated, and engaged in on-location gig work, while the self-recruited data contained more missing values.

### Measures

*Endogenous variable: Mental well-being.* Mental well-being was measured through the WHO-5 items (37): *“felt cheerful and in good spirits”, “felt calm and relaxed”, “felt active and vigorous”, “woke up feeling fresh and rested”,* and *“my life has been filled with things that interest me”* (α=0.876). Respondents rated each item with reference to the past two weeks on a 6-point Likert scale ranging from *“*all of the time*”* (1/6) to *“*at no time*”* (6/6). To reduce sparse cells, the two lowest response categories (“some of the time” and “at no time”) were combined, as the lowest category contained <10 observations (38). Mental well-being was constructed as a latent variable using confirmatory factor analysis (CFA), with factor scores derived from the model so that each item contributed according to its factor loading.

*Exogeneous variable: PE.* A version of the EPRES adapted to gig workers (EPRES-gw) (25), was used, capturing six dimensions of PE: temporariness (contractual arrangement); disempowerment (membership in a worker organization); lack of workplace rights (access to paid holiday, unemployment benefits, fixed wage, equipment cost contributions, and hospitalization insurance, α=0.793); low hourly income (average hourly wage); lack of fringe benefits (reimbursements or meal vouchers, r=0.517); and lack of training (training paid for or provided by the platform). The workplace rights dimension was adapted to the Belgian context, where social protections are largely contribution-based and linked to salaried employment, limiting access for workers outside this framework (for more information see: 39). All items were coded so that higher values indicate greater precariousness. A detailed overview of these indicators is provided in supplementary material (www.sjweh.fi/article/4278) table S1. EPRES-gw was constructed as a latent variable with factor scores derived from a CFA to reflect the contribution of each indicator according to its factor loading.

*Mediators: IQW.* Five IQW dimensions were included as mediators. Autonomy was measured with three items on control over task order, methods, and speed (α=0.585); work intensity with two items on working very fast or hard (r=0.523); physical demands with two items on heavy loads and effort (r= 0.694); skill discretion with five items on quality standards, problem-solving, task complexity, and learning opportunities (α=0.562); and social support with three items on colleagues’ interest, kindness, and assistance (α=0.849). Absence of colleagues was coded between “neither agree nor disagree” and “tend to disagree,” reflecting that having no colleagues is less beneficial than supportive colleagues but preferable to unsupportive ones; exploratory analysis of variance supported this coding (results not shown). Details are provided in supplementary table S1. The mediators were constructed as latent variables with factor scores derived from a CFA to reflect the contribution of each indicator according to its factor loading.

*Grouping variable: main vs. supplementary gig workers.* Respondents working ≤24 hours per week through the platform were categorized as supplementary gig workers, and those working >24 hours per week (the equivalent of three full workdays) as main gig workers. This approach follows prior research distinguishing gig workers by temporal engagement (35) and serves as a direct, objectively measurable indicator of exposure to PE.

### Analyses

Descriptive analyses examined means, standard deviations, and correlations among PE, IQW, and mental well-being separately for main and supplementary gig workers to assess associations and suitability for mediation. Results were deemed statistically significant if the associated P-value was <0.05.

**Figure 1:**
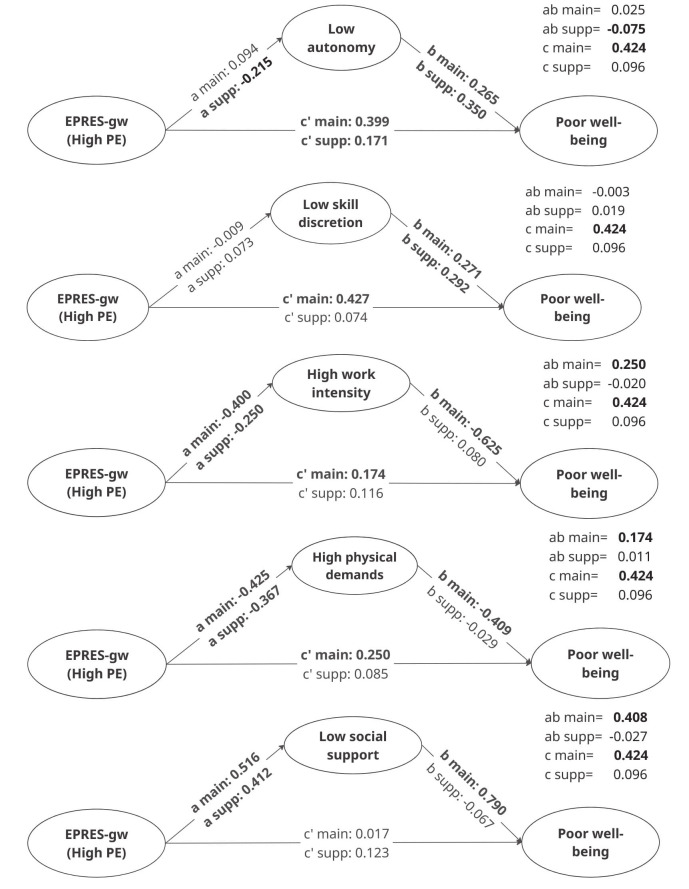
Standardized regression estimates (direct, indirect and total effects) and significance levels in the mediation model of precarious employment on poor well-being through intrinsic quality of work among main and supplementary gig workers. Source: SEAD, 2023 (own analysis). **Estimates in bold** indicate statistical significance, as zero is not within the confidence interval.

Main analyses followed a two step approach. First, measurement models were assessed using two-group CFA to generate factor scores for PE, mental well-being, and the IQW mediators by time engagement. Measurement invariance was not established (endogenous: *∆χ^2^ (**14**)=30.21, P=0.007*; exogenous: *∆χ^2^ (**10**)=35.04, P<0.001)*, preventing latent mean comparisons and requiring separate analyses by group. Nevertheless, theoretical considerations support expected differences in PE measured through the EPRES-gw, which is likely more applicable to main gig workers. Second, SEM models were estimated using the CFA-derived factor scores. Due to limited sample size, five separate models were run (one per mediator; figure 1), each including PE, mental well-being, and the mediator by time engagement. SEM allowed simultaneous estimation of direct, indirect, and total effects. Indirect effects were calculated as the product of the a and b paths, the direct effect (c′) as the remaining association between PE and mental well-being, and the total effect as their sum. Effects were estimated using 10 000 bootstrap samples with 95% percentile confidence intervals (CI). Missing data were handled using pairwise deletion.

## Results

### Descriptive results

Descriptive statistics for the sample are presented in table 1, separately for main and supplementary gig workers. Main gig workers were more likely to be younger, with a higher proportion <35 and fewer aged ≥50 years. They were also more often self-employed or engaged in temporary agency work outside their gig activities, whereas supplementary gig workers were more frequently students or outside the labor market (eg, retired, unemployed, sick leave, etc). In terms of task type, main gig workers were predominantly engaged in food delivery and transport, while supplementary gig workers more often performed other on-location tasks.

**Table 1:** Sociodemographic characteristics of the sample by main and supplementary gig workers (GW) (N=397). [NA=not available.]

Response options		Total		Main		Supplementary		χ^2^
		N (%)		N (%)		N (%)		P-value ***
Sex								
	Male	264 (66.5)		89 (69.0)		175 (65.3)		0.439
	Female	130 (32.8)		40 (31.0)		90 (33.6)		
	Other/missing/NA	3 (0.8)		0		3 (1.1)		
Age (years)								
	<35	197 (49.6)		71 (55.0)		126 (47.0)		0.002
	35–49	105 (26.5)		42 (32.6)		63 (23.5)		
	≥50	91 (22.9)		15 (11.6)		76 (28.4)		
	Other/missing/NA	4 (1.0)		1 (0.8)		3 (1.1)		
Educational level								
	Short	49 (12.3)		19 (14.7)		30 (11.2)		0.069
	Medium	114 (28.7		26 (20.2)		88 (32.8)		
	Long	224 (56.4)		80 (62.0)		144 (53.7)		
	Other/missing/NA	10 (2.5)		4 (3.1)		6 (2.2)		
Employment status outside GW								
	Employee	161 (40.6)		49 (38.0)		112 (41.8)		<0.001
	Student	61 (15.4)		9 (7.0)		52 (19.4)		
	Self-employed	65 (16.4)		35 (27.1)		30 (11.2)		
	Temp agency work	23 (5.8)		12 (9.3)		11 (4.1)		
	Outside labor market*	73 (18.4)		15 (11.6)		58 (22.1)		
	Exclusively gig job	6 (1.5)		6 (4.7)		0		
	Other/missing/NA	8 (2.0)		3 (2.3)		5 (1.9)		
Migration background								
	Native	264 (66.5)		80 (62.0)		184 (68.7)		0.097
	2^nd^ generation migrant	35 (8.8)		8 (6.2)		27 (10.1)		
	1^st^ generation migrant	92 (23.2)		38 (29.5)		54 (20.2)		
	Other/missing/NA	6 (1.5)		3 (2.3)		3 (1.1)		
Type of gig work **								
	Food delivery & transport	180 (45.3)		83 (64.3)		97 (36.2)		<0.001
	Other on-location tasks	133 (33.5)		21 (16.3)		112 (41.8)		
	Micro-tasks	17 (4.3)		1 (0.8)		16 (6.0)		
	Online professional / administrative tasks	67 (16.9)		24 (18.6)		43 (16.0)		
Data source								
	Panel	121 (30.5)		63 (48.8)		58 (21.6)		<0.001
	Survey	276 (69.5)		66 (51.2)		210 (78.4)		

Source: SEAD, 2023 (own analysis). * Gig workers who are retired, unemployed, on sick pay or homemakers. ** Initially assessed through a multiple-choice question, including predefined categories for gig work and an open-ended “other” option. Respondents could select all applicable options and then indicate their main type based on the task on which they spent the most time. These responses were subsequently re-coded into four categories: (i) food delivery and transport, (ii) other on-location tasks (eg, care work, tutoring, cleaning, handyman jobs, event work, etc), (iii) micro-tasks, and (iv) online professional and administrative tasks (e.g., writing, creative work, sales, software development, etc.). *** P-values from Chi-square tests for differences between main and supplementary gig workers.

In the full sample, PE was positively and statistically significantly associated with poorer mental well-being, although the association was modest (r=0.195). When distinguishing between main and supplementary gig workers (table 2), this relationship was substantially stronger among main gig workers (r=0.424) and not statistically significant among supplementary workers, highlighting the importance of analyzing these groups separately. PE was also statistically significantly correlated with several IQW dimensions: workers in more precarious positions reported lower physical demands (r_main_=–0.425; r_supp_=–0.367), lower work intensity (r_main_=–0.400; r_supp_=–0.250), and lower social support (r_main_=0.516; r_supp_=0.412), while associations with low skill discretion were not statistically significant, and low autonomy was negative and statistically significant only among supplementary workers (r_supp_=–0.215). In turn, IQW dimensions were related to poorer mental well-being: low skill discretion (r_main_=0.267; r_supp_=0.298) and low autonomy (r_main_=0.303; r_supp_=0.313) were statistically significant for both groups, whereas low social support (r_main_=0.799), higher physical demands (r_main_= –0.516), and greater work intensity (r_main_=–0.695) were statistically significant only for main gig workers. These patterns suggested that IQW may partially mediate the relationship between PE and mental well-being, though associations differed by time engagement.

**Table 2:** Pearson correlation (r) matrix with 95% confidence intervals (CI) for precarious employment (PE), poor well-being, and intrinsic quality of work dimensions among main (N=r129) and supplementary gig workers (N=268). **Estimates in bold** indicate statistical significance, as zero is not within the CI. Source: SEAD, 2023 (own analysis).

Variable		PE		Poor well-being		Low autonomy		High physical demands		High work intensity		Low skill discretion
		r (95% CI)		r (95% CI)		r (95% CI)		r (95% CI)		r (95% CI)		r (95% CI)
PE												
Poor well-being												
	Main	**0.424** (0.271– -0.556)										
	Supplementary	0.096 (-0.024– -0.213)										
Low autonomy												
	Main	0.094 (-0.080– -0.263		**0.303** (0.137– -0.452)								
	Supplementary	**-0.215** (-0.326– -0.098)		**0.313** (0.201– -0.417)								
High physical demands												
	Main	**-0.425** (-0.557– -0.272)		**-0.516** (-0.632– -0.377)		**-0.219** (-0.378– -0.048)						
	Supplementary	**-0.367** (-0.466– -0.259)		-0.060 (0.179–0.06)		**0.281** (0.167–0.388)						
High work intensity												
	Main	**-0.400** (-0.536– -0.244)		**-0.695** (-0.775– -0.593)		-0.181 (-0.343– -0.008)		**0.891** (0.849–0.922)				
	Supplementary	**-0.250** (-0.359– -0.134)		0.051 (-0.069–0.17)		**0.426** (0.323–0.519)		**0.688** (0.619–0.746)				
Low skill discretion												
	Main	-0.009 (-0.182– 0.164)		**0.267** (0.099–0.420)		**0.916** (0.883–0.94)		-0.080 (-0.249–0.094)		-0.180 (-0.342– -0.007)		
	Supplementary	0.073 (-0.047–0.191)		**0.298** (0.185–0.403)		**0.550** (0.461–0.628)		**-0.250** (-0.359– -0.134)		**-0.322** (-0.425– -0.210)		
Low social support												
	Main	**0.516** (0.377–0.632)		**0.799** (0.726–0.854)		0.268 (-0.100–0.421)		**-0.654** (-0.743– -0.542)		**-0.621** (-0.717–-0.502)		0.075 (0.099–0.245)
	Supplementary	**0.412** (0.307–0.507)		-0.016 (-0.136– 0.104]		**-0.256** (-0.365– -0.140)		**-0.401** (-0.497– -0.295)		**-0.187** (-0.300– -0.069)		0.123 (0.003–0.239)

### SEM results

First, we examined the overall relationship between PE and poor mental well-being, investigating whether this relationship was stronger among main gig workers (table 3) than supplementary gig workers (table 4). We compared the total effects of PE. For both groups, total effects were the same across all five mediation models. The standardized coefficient for main gig workers was positive and statistically significant (b=0.424) but was not significant for supplementary workers.

**Table 3:** Standardized regression estimates and confidence intervals (CI) of five structural models of poor well-being, precarious employment and intrinsic quality of work for **main gig workers** (N=129). Source: SEAD, 2023 (own analysis). **Estimates in bold** indicate statistical significance, as zero is not within the CI. [SE=standard error.]

		Low autonomy		Low skill discretion		High work intensity		High physical demands		Low social support
		b (SE) (95% CI)		b (SE) (95% CI)		b (SE) (95% CI)		b (SE) (95% CI)		b (SE) (95% CI)
**Direct effects (c’)**										
Precarious employment										
	*Mediator*	0.094 (0.105) (-0.091–0.324)		-0.009 (0.018) (-0.037–0.033)		**-0.400** (0.112) (-0.744– -0.308)		**-0.425** (0.108) (-0.748– -0.326)		**0.516** (0.099) (0.560–0.952)
	*Poor mental well-being*	**0.399** (0.098) (0.342–0.730)		**0.427** (0.095) (0.387–0.758)		**0.174** (0.101) (0.037–0.429)		**0.250** (0.114) (0.105–0.559)		0.017 (0.102) (-0.172–0.225)
Low autonomy										
	*Poor mental well-being*	**0.265** (0.089) (0.106–0.457)								
Low skill discretion										
	*Poor mental well-being*			**0.271** (0.531) (0.724–2.792						
High work intensity										
	*Poor mental well-being*					**-0.625** (0.080) (-0.785– -0.472)				
High physical demands										
	*Poor mental well-being*							**-0.409** (0.097) (-0.619– -0.240)		
Low social support										
	*Poor mental well-being*									**0.790** (0.063) (0.591–0.833)
**Indirect effects (ab)**										
Precarious employment										
	*Poor mental well-being*	0.025 (0.032) (-0.028–0.101)		-0.003 (0.033) (-0.076–0.058)		**0.250** (0.088) (0.178–0.519)		**0.174** (0.074) (0.107–0.392)		**0.408** (0.089) (0.378–0.731)
**Total effects (c)**										
Precarious employment										
	*Poor mental well-being*	**0.424** (0.104) (0.362–0.771)		**0.424** (0.104) (0.362–0.771)		**0.424** (0.104) (0.362–0.771)		**0.424** (0.104) (0.362–0.771)		**0.424** (0.104) (0.362–0.771)

**Table 4:** Standardized regression estimates and confidence intervals (CI) of five structural models of poor well-being, precarious employment and intrinsic quality of work for **supplementary gig workers** (N=268). Source: SEAD, 2023 (own analysis). **Estimates in bold** indicate statistical significance, as zero is not within the CI. [SE=standard error.]

		Low autonomy		Low skill discretion		High work intensity		High physical demands		Low social support
		b (SE) (95% CI)		b (SE) (95% CI)		b (SE) (95% CI)		b (SE) (95% CI)		b (SE) (95% CI)
**Direct effects (c’)**										
Precarious employment										
	*Mediator*	**0.215** (0.084) (-0.470– -0.142)		0.073 (0.017) (-0.013–0.052)		**-0.250** (0.089) (-0.561– -0.220)		**-0.367** (0.110) (-0.878– -0.456)		**0.412** (0.097) (0.523–0.893)
	*Poor mental well-being*	**0.171** (0.113) (0.095–0.537)		0.074 (0.112) (-0.080–0.356)		0.116 (0.121) (-0.034–0.458)		0.085 (0.130) (-0.105–0.416)		0.123 (0.130) (-0.035–0.490)
Low autonomy										
	*Poor mental well-being*	**0.350** (0.075) (0.313–0.610)								
Low skill discretion										
	*Poor mental well-being*			**0.292** (0.378) (1.210—2.687)						
High work intensity										
	*Poor mental well-being*					0.080 (0.082) (-0.064–0.255)				
High physical demands										
	*Poor mental well-being*							-0.029 (0.073) (-0.173–0.110)		
Low social support										
	*Poor mental well-being*									-0.067 (0.066) (-0.204–0.059)
**Indirect effects (ab)**										
Precarious employment										
	*Poor mental well-being*	**-0.075** (0.045) (-0.237– -0.060)		0.019 (0.035) (-0.026–0.109)		-0.020 (0.033) (-0.102–0.027)		0.011 (0.051) (-0.073–0.122)		-0.027 (0.047) (-0.146–0.043)
**Total effects (c)**										
Precarious employment										
	*Poor mental well-being*	0.096 (0.117) (-0.060—0.412))		0.096 (0.117) (-0.060—0.412))		0.096 (0.117) (-0.060—0.412))		0.096 (0.117) (-0.060—0.412))		0.096 (0.117) (-0.060—0.412))

Second, we examined whether there were positive and statistically significant mediation effects of the five IQW indicators and determined whether these effects were stronger for main versus supplementary gig workers. We discuss each of the five mediation models accordingly (figure 1, tables 3 and 4).

For low autonomy, no mediation was observed for main gig workers. In contrast, supplementary workers showed a statistically significant negative indirect effect (b= -0.075, proportion mediated=78.6%), indicating partial mediation. The a-path was negative for supplementary workers (b= -0.215), meaning that higher PE scores were associated with higher autonomy scores. The b-paths were positive and statistically significant in both groups (b=0.265 and 0.350), showing that lower autonomy was linked to poorer mental well-being. Controlling for autonomy, the direct effect of PE remained statistically significant for both groups (b=0.399 and 0.171) even though the total effect for supplementary workers was not statistically significant. This implied that low autonomy had a suppressing effect on the relationship between PE and poor mental well-being among supplementary workers.

For low skill discretion, no statistically significant indirect effects were found in either group. The a-paths were not statistically significant, showing no direct association between PE and skill discretion, whereas the b-paths were statistically significant for both groups (b=0.271 and 0.292). Thus, while we found no evidence of a mediating role for skill discretion, we did find that it explained part of the variation in the mental well-being of gig workers. Controlling for skill discretion, the direct effect remained statistically significant for main but not supplementary workers (b=0.427).

In the high work intensity model, main gig workers showed a positive, significant indirect effect (b=0.250) while supplementary workers did not. The a-paths were statistically significant but negative for both groups (b= -0.400 and -0.250), indicating that high scores on PE were associated with lower work intensity. The b-paths indicated a statistically significant negative effect only for main gig workers, where high work intensity (b= -0.625) was associated with better mental well-being scores, opposite to the hypothesized direction. Controlling for work intensity, the direct effect of PE for main gig workers decreased compared to the total effect but remained statistically significant (b=0.174) indicating partial mediation (proportion mediated=58.9%), while it was not statistically significant for supplementary workers.

In the mediation model with high physical demands, a positive, statistically significant indirect effect was observed only for main gig workers (b=0.174). The a-paths were statistically significant but negative for both groups (b= -0.425 and -0.367), while the b-path was negative and significant only for main gig workers (b= -0.409), indicating better mental well-being with higher physical demands. Controlling for high physical demands, the direct effect of PE on poor mental well-being remained statistically significant for main gig workers (b=0.250), indicating partial mediation (proportion mediated=41.0%), whereas it was not statistically significant for supplementary workers.

Finally, in the model with low social support, main gig workers showed a positive, statistically significant indirect effect (b=0.408) whereas this effect was not statistically significant for supplementary workers. The a-paths were positive and statistically significant for both groups (b=0.516 and 0.412), showing that high scores on PE were linked to lower social support. The b-path was positive and statistically significant only for main gig workers (b=0.790) indicating that lower social support was associated with poorer mental well-being in this group. Controlling for low social support, the direct effect of PE on mental well-being became not statistically significant for main gig workers, marking the only model with full mediation (proportion mediated=96.1%). No mediation was observed for supplementary workers.

Sensitivity analyses using linear regression models showed that covariate adjustment did not materially alter the results, except for counterintuitive effects for work intensity and physical demands (see supplementary tables S2–5).

## Discussion

This study examined the relationship between PE and gig workers’ mental well-being, the mediating role of IQW and differences between main and supplementary gig workers, employing SEM.

Our results confirmed a statistically significant relationship between high PE and poor mental well-being for main but not supplementary gig workers. This relationship remained robust across most mediation models, except in the low social support model where full mediation occurred. These findings contrast with prior EPRES studies suggesting that IQW largely mitigates the impact of PE [eg (9),]. The absence of an association for supplementary workers (except in the low autonomy model) suggests that PE poses greater mental health risks for main gig workers, which is consistent with previous research (12).

Mediation through IQW varied by time spent in gig work. Social support was particularly important for main gig workers, whereas autonomy played a more prominent role for supplementary workers. For the latter, high PE was associated with higher levels of autonomy, but higher levels of autonomy were associated with better mental well-being. Platform-promoted narratives suggesting that flexibility enhances autonomy appear to hold (12) however, controlling for autonomy revealed an association between high PE and poor mental well-being, indicating that PE remains harmful even when workers benefit from flexibility. These findings highlight the importance of fostering autonomy alongside efforts to reduce PE.

For main gig workers, PE’s association with mental well-being was partially mediated by work intensity, high physical demands, and low social support. Mediation via low social support fully accounted for the association between PE and mental well-being, highlighting its critical role. Highly precarious gig jobs are characterized by weaker social support, reflecting individualized and competitive work arrangements (6), and often limited face-to-face interaction (29). This erosion of social support increases social isolation, which undermines mental well-being (6). Mediation via physical demands and work intensity appeared counterintuitive, as higher PE was associated with lower physical demands and lower work intensity. Sensitivity analyses using simplified linear regressions – each including a single predictor, a covariate, and a single outcome – showed that controlling for age and an age-intensity interaction rendered these effects non-significant for main gig workers. For supplementary workers, controlling for age eliminated the association between high work intensity and PE and controlling for gender eliminated the association with high physical demands. These results suggest that apparent links between PE, physical demands, and work intensity largely reflect sociodemographic differences, including occupational gender segregation, highlighting the need to account for age and gender in analyses.

Among main gig workers, high work intensity and high physical demands were associated with better mental well-being. Controlling for education removed the association with physical demands, but the association with high work intensity remained robust even after adjusting for sociodemographic variables, income, job type, job satisfaction, and work motivation. This may reflect differences in how workers interpret and cope with PE (40). Some perceive gig work as a temporary, aspirational step in a broader career trajectory or as an entrepreneurial opportunity, framing demanding work as productive and legitimate (41, 42). These subjective processes vary: workers with fewer alternatives may positively reinterpret PE, while others resist or exit, producing a ‘survivor effect’ (41). This could explain why the negative association with high work intensity appears only for main gig workers who are the most exposed to both the work and normalization of precarity. This does not imply that only ‘stronger’ workers survive; health effects likely exist, but subjectification may obscure the PE–mental health relationship. Further research is needed to clarify whether these patterns reflect selective survival or hidden health risks.

Several limitations should be noted. First, the sample (N=397) is modest relative to the complexity of the analyses. This limited covariate inclusion and necessitated separate mediation models rather than a single fully specified model. Some associations may partly reflect differences in age, gender, education, or other background factors, and models do not control for multiple mediators simultaneously. Sensitivity analyses using linear regressions indicated that controlling for covariates did not alter results, except for counterintuitive effects of work intensity and physical demands. Bootstrapped mediation estimates further supported the stability of findings. Nevertheless, given the sample size and number of models, results should be interpreted with caution. At the same time, larger and representative surveys, such as the Collaborative Economy and Employment (COLLEEM) (43) and Internet and Platform Work surveys (44) do not capture this level of detailed job quality data, making our sample among the first to provide such measures (27). Second, the cross-sectional design precludes causal inference; mediation estimates should be interpreted as statistical associations rather than causal pathways. Third, generalizability is limited. Comparisons with similar surveys suggest that the sample broadly reflects the population, though self-employed, salaried, and online workers are slightly underrepresented, while students, undocumented migrants, and individuals outside the labor force are overrepresented (36). Fourth, the main (>24 hour/week) versus supplementary (≤24 hours/week) distinction relies on a time threshold that requires further validation (16). While our operationalization aligns with our conceptual focus on exposure to PE, future research could refine this classification using time engagement, economic dependency [eg, income share from gig work, multiple job holding (16, 45)] and household-level resources.

### Concluding remarks

This study demonstrated that higher PE was associated with poorer mental well-being among gig workers, particularly among those who spend significant time in PE. IQW partially mediated this association. Among main gig workers, low social support fully explained the PE–mental well-being link, highlighting risks of social isolation. Among supplementary workers, high PE showed a weaker association with poor mental well-being, whereas low autonomy and low skill discretion were more strongly associated with poorer mental well-being. While regulations enhancing social rights, contractual security, and collective representation such as the European Platform Work Directive provide a foundation, traditional psychosocial risks linked to IQW remain critical. Interventions and research must differentiate between main and supplementary gig workers to address their distinct occupational health risks.

## Supplementary material

Supplementary material

## Data Availability

Unfortunately, the data supporting the findings of this study are not publicly available because our respondents did not consent to the disclosure of their data in a public repository. It is not possible to seek this consent retrospectively.
