# Supplementary material for: Precarious employment and its relation to mental well-being in the gig economy: comparing main and supplementary workers
Source: Scand J Work Environ Health. 2026 Apr 30;52(3):241–51. doi: 10.5271/sjweh.4278 (PMC13181860; doi:10.5271/sjweh.4278)
Supplement: Supplementary material [file SJWEH-52-241-S001.pdf]

Precarious employment and its relation to mental well-being in the gig economy: comparing main and supplementary workers[1]

by Elief Vandevenne,[2] Christophe Vanroelen, Lara Stas, Jessie Gevaert

1. Supplementary material
2. Correspondence to: Elief Vandevenne, Brussels Institute for Social and Population Studies (BRISPO), Vrije Universiteit Brussel, Belgium. [E-mail: Elief.Vandevenne@vub.be] https://orcid.org/0000-0002-8700-5511

Table S1 Operationalization of precarious employment and intrinsic quality of work for Belgian gig workers.

	**Dimension of EPRES-gw**	**Content**	**Indicators (= α)**	**Response options and coding**
**Precarious Employment**	Temporariness	Type of contract when working for platform	Under what type of contract do you work for the platform {name of platform}?	1. Employee open-ended<br>2. Temporary contract /Student<br>3. Self-employed/Peer-2-Peer/Interim/Self-employed student/Flexi job<br>4. No contract
	Disempowerment	No worker representation	Are you a member of an organization that defends your interests? (multiple responses possible)	0. Yes (of a union/collective/cooperative/self-employment organization/other)<br>1. No
	Lack of Rights	No access to workplace rights	I am entitled to paid holiday/ unemployment benefits/ fixed wage/ contribution to equipment costs/ hospitalization insurance (α = 0.793)	0. Yes<br>1. No
	Low Hourly Income	Low hourly wage from gig job	If you had to express your wage per hour, how much would you earn on average?	Numeric value
	Lack of Fringe Benefits	No access to extra benefits	I am entitled to fringe benefits/reimbursements (r = 0.517)	0. Yes<br>1. No
	Lack of Training	No skill development opportunities	Since you started gig work, have you received training paid for or provided by the platform?	0. Yes<br>1. No
**Intrinsic Quality of Work**	Low Autonomy	Little control over how/when work is done	Can you choose/change: task order, methods, speed? (α = 0.585)	0. Yes<br>1. No
	High Work Intensity	Fast pace or heavy workload	My job requires me to work really fast/hard (r=0.523)	1. Strongly disagree<br>2. Tend to disagree<br>3. Neutral<br>4. Tend to agree<br>5. Strongly agree

	High Physical Demands	Strenuous physical tasks	My job requires me to carry heavy loads/ use physical effort (r=0.694)	1. Strongly disagree<br>2. Tend to disagree<br>3. Neutral<br>4. Tend to agree<br>5. Strongly agree
	Low Skill discretion	Little opportunity to use or develop skills	My tasks involve: meeting precise quality standards/ self-assessing the quality of my work/ solving unforeseen problems/ learning new things (α = 0.562)	0. Yes<br>1. No
	Low Social Support	Low support from colleagues	My colleagues take an interest in me/ are kind/ help me to complete my work (α = 0.849)	1. Strongly agree<br>2. Tend to agree<br>3. Neither agree, nor disagree<br>4. I don’t have colleagues<br>5. Tend to disagree<br>6. Strongly disagree

Table S2 Sensitivity analysis of physical demands, work intensity, well-being, and precarious employment: linear regression results.

Worker type	Outcome	Predictor	Control variable	Estimate	S.E.	P-value	Interaction	Implication
Main	PE	High work intensity	Age	0.271	0.220	0.221	INT × Age = -0.017, p = 0.007	High-intensity work linked to higher PE for younger workers; effect decreases with age
Main	PE	Age	—	-0.002	0.004	0.517	—	
Main	PE	High work intensity	Education	-0.117	0.215	0.589	INT × Education = - NS	Effect of intensity on PE not significant after controlling for education
Main	PE	Education	—	-0.154	0.053	0.004	—	
Supp	PE	High work intensity	Work type	-0.027	0.065	0.676	INT × Work type NS	Negative association disappears when controlling for work type
Supp	PE	Work type	—	0.302–0.337	0.058–0.067	<0.001	—	
Supp	PE	High work intensity	Age	-0.128	0.103	0.216	INT × Age NS	Negative association disappears when controlling for age
Supp	PE	Age	—	0.004	0.002	0.009	—	
Main	PE	High physical demands	Age	0.247	0.225	0.273	PHYS × Age = -0.016, p = 0.007	Physical demands increase PE for younger workers; effect decreases with age
Main	PE	Age	—	-0.006	0.004	0.152	—	
Main	PE	High physical demands	Gender	-0.220	0.189	0.248	PHYS × Gender = - NS	Effect not significant after controlling for gender
Main	PE	Gender	—	-0.192	0.086	0.028	—	
Main	PE	High physical demands	Education	-0.370	0.222	0.098	PHYS × Education NS	Effect not significant after controlling for education
Main	PE	Education	—	-0.188	0.052	0.000	—	
Supp	PE	High physical demands	Gender	-0.100	0.095	0.296	PHYS × Gender NS	Negative association disappears when controlling for gender
Supp	PE	Gender	—	0.024	0.052	0.646	—	
Main	WHO	High work intensity	High enjoyment	-0.797	0.076	<0.001	INT × Enjoyment = 0.302, p = 0.026	Intensity reduces poor well-being more strongly when enjoyment is low
		High enjoyment	—	-0.191	0.084	<0.05		
Main	WHO	High work intensity	IQW mediators	-1.141	0.125	<0.001	—	Robust negative association with poor mental well-being, controlling for all IQW mediators
Main	WHO	High work intensity	All sociodemographic variables	-0.690	0.069	<0.001	—	Robust negative association with poor mental well-being, controlling for work type, gender, education, citizenship, age
Main	WHO	High physical demands	Gender	-0.396	0.244	0.106	PHYS × Gender NS	Effect not significant after controlling for gender

Worker type	Outcome	Predictor	Control variable	Estimate	S.E.	P-value	Interaction	Implication
		Gender	—	-0.048	0.111	0.667	—	
Main	WHO	High physical demands	Education	-0.461	0.285	0.109	PHYS × Education NS	Effect not significant after controlling for education
		Education	—	-0.133	0.067	<0.05	—	

Source: SEAD, 2023 (own analysis).

Table S3 Sensitivity analyses of standardized mean scores for precarious employment, poor mental well-being, and intrinsic quality of work by sociodemographic characteristics, gig-work type, and time spent in gig work among Belgian gig workers (n = 376)

	**Precarious Employment**		**Poor well-being**		**Low autonomy**		**High work intensity**		**High physical demands**		**Low skill discretion**		**Low social support**	
	Mean (S.D.)		Mean (S.D.)		Mean (S.D.)		Mean (S.D.)		Mean (S.D.)		Mean (S.D.)		Mean (S.D.)	
	Supp	Main	Supp	Main	Supp	Main	Supp	Main	Supp	Main	Supp	Main	Supp	Main
**Gender (⸙)** (Sig.)	p=0.075	p=0.113	p=0.356	p=0.668	p=0.091	p=0.119	**p=0.009**	p=0.241	**p=0.034**	p=0.210	p=0.294	p=0.108	p=0.136	p=0.761
Male	0.585 (0.209)	0.544 (0.255)	0.478 (0.202)	0.484 (0.178)	0.432 (0.214)	0.424 (0.218)	0.557 (0.198)	0.550 (0.195)	0.481 (0.218)	0.479 (0.173)	0.413 (0.243)	0.415 (0.216)	0.467 (0.193)	0.474 (0.196)
Female	0.638 (0.226)	0.424 (0.218)	0.504 (0.217)	0.498 (0.165)	0.391 (0.163)	0.368 (0.167)	0.486 (0.206)	0.503 (0.201)	0.419 (0.218)	0.434 (0.188)	0.444 (0.214)	0.356 (0.167)	0.505 (0.188)	0.485 (0.192)
**Age** (⸙⸙) (Sig.)	**p=0.014**	**p<0.001**	**p<0.001**	p=0.180	**p<0.001**	p=0.229	**p=0.005**	p=0.054	p=0.120	**p=0.003**	**p<0.001**	**p=0.028**	**p=0.002**	**p=0.016**
Younger than 35	0.561 (0.224)	0.541 (0.253)	0.531 (0.195)	0.510 (0.180)	0.489 (0.196)	0.434 (0.201)	0.577 (0.209)	0.517 (0.193)	0.486 (0.240)	0.456 (0.166)	0.468 (0.239)	0.428 (0.207)	0.436 (0.210)	0.477 (0.174)
35–49	0.633 (0.207)	0.413 (0.216)	0.515 (0.202)	0.448 (0.174)	0.425 (0.187)	0.388 (0.207)	0.503 (0.175)	0.589 (0.205)	0.416 (0.207)	0.521 (0.182)	0.444 (0.228)	0.391 (0.197)	0.503 (0.155)	0.434 (0.210)
50 and older	0.646 (0.200)	0.725 (0.223)	0.393 (0.203)	0.507 (0.123)	0.301 (0.155)	0.342 (0.200)	0.488 (0.205)	0.460 (0.165)	0.454 (0.189)	0.346 (0.164)	0.335 (0.206)	0.274 (0.160)	0.530 (0.174)	0.600 (0.187)
**Level of education** (⸙⸙) (Sig.)	p=0.944	**p=0.002**	p=0.557	p=0.154	p=0.881	p=0.766	p=0.763	p=0.954	**p=0.030**	p=0.340	p=0.651	p=0.960	p=0.237	p=0.464
Primary	0.603 (0.255)	0.572 (0.282)	0.502 (0.236)	0.490 (0.177)	0.431 (0.218)	0.392 (0.195)	0.554 (0.226)	0.587 (0.179)	0.497 (0.255)	0.524 (0.167)	0.422 (0.238)	0.380 (0.194)	0.517 (0.161)	0.483 (0.216)
Secondary	0.601 (0.219)	0.688 (0.209)	0.492 (0.219)	0.563 (0.201)	0.414 (0.189)	0.407 (0.246)	0.527 (0.182)	0.471 (0.212)	0.497 (0.202)	0.440 (0.164)	0.438 (0.230)	0.414 (0.231)	0.484 (0.191)	0.514 (0.222)
Tertiary	0.604 (0.207)	0.452 (0.236)	0.480 (0.195)	0.463 (0.157)	0.419 (0.202)	0.410 (0.193)	0.533 (0.212)	0.545 (0.194)	0.431 (0.219)	0.460 (0.184)	0.415 (0.236)	0.394 (0.197)	0.470 (0.198)	0.464 (0.179)
**Migration background** (⸙⸙) (Sig.)	p=0.133	**p=0.018**	**p=0.033**	**p=0.015**	**p<0.001**	**p=0.030**	**p<0.001**	p=0.071	**p=0.008**	p=0.120	p=0.792	p=0.101	**p=0.019**	p=0.140
Native	0.616 (0.215)	0.476 (0.252)	0.468 (0.200)	0.457 (0.168)	0.392 (0.190)	0.376 (0.183)	0.508 (0.195)	0.561 (0.203)	0.436 (0.200)	0.486 (0.192)	0.426 (0.237)	0.373 (0.183)	0.496 (0.183)	0.453 (0.200)
Second-generation migrant	0.586 (0.223)	0.621 (0.316)	0.530 (0.237)	0.581 (0.155)	0.444 (0.206)	0.444 (0.262)	0.559 (0.211)	0.471 (0.135)	0.521 (0.267)	0.404 (0.100)	0.411 (0.240)	0.427 (0.287)	0.474 (0.183)	0.589 (0.117)
First-generation migrant	0.565 (0.215)	0.591 (0.228)	0.532 (0.213	0.536 (0.175)	0.503 (0.205)	0.463 (0.224)	0.614 (0.211)	0.493 (0.192)	0.519 (0.247)	0.433 (0.156)	0.418 (0.222)	0.439 (0.220)	0.422 (0.220)	0.505 (0.186)

**Type of PW** (⸙⸙) (Sig.)	**p<0.001**	p=0.947	p=0.245	**p=0.026**	**p<0.00 1**	p=0.059	**p=0.003**	**p=0.010**	**p<0.001**	p=0.088	**p=0.002**	p=0.055	**p<0.001**	**p=0.010**
Food delivery couriers and ride-hailers	0.483 (0.206)	0.508 (0.244)	0.509 (0.198)	0.470 (0.175)	0.563 (0.191)	0.444 (0.212)	0.620 (0.187)	0.572 (0.186)	0.567 (0.222)	0.490 (0.175)	0.490 (0.234)	0.437 (0.205)	0.378 (0.203)	0.441 (0.186)
Other on-location tasks	0.672 (0.190)	0.592 (0.274)	0.438 (0.205)	0.476 (0.169)	0.334 (0.158)	0.312 (0.184)	0.476 (0.192)	0.485 (0.180)	0.441 (0.185)	0.412 (0.167)	0.385 (0.224)	0.286 (0.196)	0.526 (0.175)	0.534 (0.224)
Micro tasks	0.774 (0.147)	0.170 (NA)	0.513 (0.230)	0.418 (NA)	0.432 (0.165)	0.395 (NA)	0.425 (0.165)	0.599 (NA)	0.231 (0.107)	0.570 (NA)	0.546 (0.213)	0.452 (NA)	0.621 (0.057)	0.382 (NA)
Online professional services	0.623 (0.195)	0.512 (0.268)	0.555 (0.205)	0.565 (0.163)	0.314 (0.121)	0.363 (0.165)	0.532 (0.215)	0.454 (0.227)	0.364 (0.207)	0.421 (0.189)	0.327 (0.210)	0.355 (0.167)	0.528 (0.137)	0.555 (0.167)

***Source**:* SEAD, 2023 (own analysis). Sig. = significance level; Main = main gig workers (N=255); Supp = supplementary gig workers (N=121); ⸙ = t-test; ⸙⸙ = ANOVA.

Table S4 Sensitivity analyses using stepwise linear regression models of precarious employment predicting mental well-being with dimensions of intrinsic quality of work (low autonomy, high work intensity, low skill discretion, low social support, and high physical demands) and sociodemographic among **main gig workers** (n = 121)

	**Model 1:** Low autonomy			**Model 2:** High work intensity			**Model 3:** High physical demands			**Model 4:** Low Skill discretion			**Model 5:** Low social support		
	(1)	(2)	(3)	(1)	(2)	(3)	(1)	(2)	(3)	(1)	(2)	(3)	(1)	(2)	(3)
	B (SD)<br>*p-value*	*B (SD)*<br>*p-value*	*B (SD)*<br>*p-value*	*B (SD)*<br>*p-value*	*B (SD)*<br>*p-value*	*B (SD)*<br>*p-value*	*B (SD)*<br>*p-value*	*B (SD)*<br>*p-value*	*B (SD)*<br>*p-value*	*B (SD)*<br>*p-value*	*B (SD)*<br>*p-value*	*B (SD)*<br>*p-value*	*B (SD)*<br>*p-value*	*B (SD)*<br>*p-value*	*B (SD)*<br>*p-value*
**Intercept**	-0.025<br>(0.054)<br>p=0.653	-0.030<br>(0.053)<br>p= 0.570	-0.050<br>(0.091)	-0.025<br>(0.054)<br>p=0.653	-0.014<br>(0.041)<br>p=0.741	-0.027<br>(0.071)<br>p=0.708	-0.025<br>(0.054)<br>p=0.653	-0.023<br>(0.050)<br>p=0.648	-0.026<br>(0.085)<br>p=0.760	-0.025<br>(0.054)<br>p=0.653	-0.029<br>(0.053)<br>p=0.583	-0.061<br>(0.091)<br>p=0.503	-0.025<br>(0.054)<br>p=0.653	-0.007<br>(0.036)<br>p=0.843	0.036<br>(0.060)<br>p=0.554
**PE**	**0.516**<br>(0.109)<br>p<0.001	**0.499**<br>(0.106)<br>p< 0.001	**0.464**<br>(0.120)<br>p< 0.001	**0.516**<br>(0.109)<br>p<0.001	0.156<br>(0.091)<br>p=0.090	0.162<br>(0.099)<br>p=0.106	**0.516**<br>(0.109)<br>p<0.001	**0.274**<br>(0.110)<br>p=0.015	**0.280**<br>(0.118)<br>p=0.020	**0.516**<br>(0.109)<br>p<0.001	**0.531**<br>(0.106)<br>p<0.001	**0.480**<br>(0.120)<br>p<0.001	**0.516**<br>(0.109)<br>p<0.001	0.010<br>(0.083)<br>p=0.905	0.019<br>(0.087)<br>p=0.824
**Low autonomy**		**0.267**<br>(0.087)<br>p= 0.003	**0.241**<br>(0.089)<br>p=0.008												
**High work intensity**					**-0.667**<br>(0.071)<br>p<0.001	**-0.659**<br>(0.071)<br>p<0.001									
**High physical demands**								**-0.440**<br>(0.087)<br>p<0.001	**-0.448**<br>(0.088)<br>p<0.001						
**Low skill discretion**											**1.613**<br>(0.522)<br>p=0.003	**1.474**<br>(0.543)<br>p=0.008			
**Low social support**														**0.707**<br>(0.057)<br>p<0.001	**0.720**<br>(0.056)<br>p<0.001
**Age** (ref.= <35)															
35-49			-0.056<br>(0.119)<br>p=0.640			-0.017<br>(0.093)<br>p=0.854			-0.035<br>(0.111)<br>p=0.755			-0.054<br>(0.119)<br>p=0.652			-0.096<br>(0.078)<br>p=0.224
>=50			-0.067<br>(0.183)<br>p=0.715			-0.157<br>(0.142)<br>p=0.270			-0.245<br>(0.172)<br>p=0.157			-0.018<br>(0.186)<br>p=0.925			**-0.318**<br>(0.121)<br>p=0.010
**Migration background** (ref.= native)															
Second-generation migrant			0.270<br>(0.219)<br>p=0.218			0.211<br>(0.170)<br>p=0.217			0.234<br>(0.204)<br>p=0.254			0.278<br>(0.218)<br>p=0.205			0.059<br>(0.145)<br>p=0.683
First-generation migrant			0.109<br>(0.129)<br>p=0.398			0.081<br>(0.099)<br>p=0.416			0.100<br>(0.119)<br>p=0.404			0.129<br>(0.128)<br>p=0.314			0.083<br>(0.084)<br>p=0.323
**R^2**	0.158	0.221	0.238	0.158	0.521	0.538	0.158	0.308	0.338	0.158	0.221	0.238	0.158	0.635	0.669
**Adj. R^2**	0.151	0.207	0.198	0.151	0.512	0.513	0.151	0.296	0.303	0.151	0.207	0.198	0.151	0.629	0.652

Source: SEAD, 2023 (own analysis). PE= precarious employment.

Table S5 Sensitivity analyses using stepwise linear regression models of precarious employment predicting mental well-being with dimensions of intrinsic quality of work (low autonomy, high work intensity, low skill discretion, low social support, and high physical demands) and sociodemographic among **supplementary gig workers** (n=255).

	**Model 1:** Low autonomy			**Model 2:** High work intensity			**Model 3:** High physical demands			**Model 4:** Low Skill discretion			**Model 5:** Low social support		
	(1)	(2)	(3)	(1)	(2)	(3)	(1)	(2)	(3)	(1)	(2)	(3)	(1)	(2)	(3)
	B (SD)<br>*p-value*	*B (SD)*<br>*p-value*	*B (SD)*<br>*p-value*	*B (SD)*<br>*p-value*	*B (SD)*<br>*p-value*	*B (SD)*<br>*p-value*	*B (SD)*<br>*p-value*	*B (SD)*<br>*p-value*	*B (SD)*<br>*p-value*	*B (SD)*<br>*p-value*	*B (SD)*<br>*p-value*	*B (SD)*<br>*p-value*	*B (SD)*<br>*p-value*	*B (SD)*<br>*p-value*	*B (SD)*<br>*p-value*
**Intercept**	-0.019<br>(0.055)<br>p=0.724	-0.079<br>(0.054)<br>p=0.143	0.001<br>(0.081)<br>p=0.987	-0.019<br>(0.055)<br>p=0.724	-0.026<br>(0.055)<br>p=0.639	0.070<br>(0.080)<br>p=0.385	-0.019<br>(0.055)<br>p=0.724	-0.013<br>(0.056)<br>p=0.816	0.076<br>(0.080)<br>p=0.344	-0.019<br>(0.055)<br>p=0.724	-0.040<br>(0.053)<br>p=0.456	0.020<br>(0.080)<br>p=0.804	-0.019<br>(0.055)<br>p=0.724	-0.028<br>(0.056)<br>p=0.612	0.071<br>(0.081)<br>p=0.381
**PE**	0.095<br>(0.115)<br>p=0.409	**0.244**<br>(0.113)<br>p=0.032	**0.265**<br>(0.112)<br>p=0.019	0.095<br>(0.115)<br>p=0.409	0.134<br>(0.120)<br>p=0.265	0.198<br>(0.115)<br>p=0.088	0.095<br>(0.115)<br>p=0.409	0.055<br>(0.125)<br>p=0.661	0.143<br>(0.121)<br>p=0.239	0.095<br>(0.115)<br>p=0.409	0.068<br>(0.112)<br>p=0.544	0.155<br>(0.110)<br>p=0.162	0.095<br>(0.115)<br>p=0.409	0.143<br>(0.128)<br>p= 0.265	0.192<br>(0.123)<br>p=0.121
**Low autonomy**		**0.420**<br>(0.081)<br>p<0.001	**0.295**<br>(0.087)<br>p=0.001												
**High work intensity**					0.090<br>(0.076)<br>p=0.240	0.024<br>(0.075)<br>p=0.753									
**High physical demands**								-0.055<br>(0.068)<br>p=0.416	-0.067<br>(0.066)<br>p=0.310						
**Low skill discretion**											**1.740**<br>(0.406)<br>p<0.001	**1.346**<br>(0.407)<br>p=0.001			
**Low social support**														-0.064<br>(0.075)<br>p= 0.393	-0.003<br>(0.073)<br>p=0.963
**Age** (ref.= <35)															
35-49			-0.027<br>(0.116)<br>p=0.818			-0.060<br>(0.119)<br>p=0.613			-0.072<br>(0.119)<br>p=0.542			-0.041<br>(0.116)<br>p=0.724			-0.063<br>(0.119)<br>p=0.594
>=50			**-0.372**<br>(0.117)<br>p=0.002			**-0.509**<br>(0.112)<br>p<0.001			**-0.511**<br>(0.111)<br>p<0.001			**-0.417**<br>(0.113)<br>p<0.001			**-0.513**<br>(0.113)<br>p<0.001
**Migration background** (ref.= native)															
Second-generation migrant			0.161<br>(0.149)<br>p=0.282			0.184<br>(0.153)<br>p=0.230			0.205<br>(0.153)<br>p=0.183			0.206<br>(0.150)<br>p=0.170			0.187<br>(0.153)<br>p=0.222
First-generation migrant			0.113<br>(0.121)<br>p=0.350			0.173<br>(0.124)<br>p=0.164			0.194<br>(0.123)<br>p=0.115			0.197<br>(0.119)<br>p=0.101			0.178<br>(0.123)<br>p=0.147
**R^2**	0.003	0.099	0.147	0.003	0.008	0.107	0.003	0.005	0.111	0.003	0.071	0.145	0.003	0.006	0.107
**Adj. R^2**	-0.001	0.092	0.126	-0.001	0.000	0.086	-0.001	-0.003	0.089	-0.001	0.063	0.124	-0.001	-0.002	0.085

***Source**:* SEAD, 2023 (own analysis). PE= precarious employment.
